# Associations between Physical Activity Trajectories and Incident Hypertension

**DOI:** 10.31083/j.rcm2311385

**Published:** 2022-11-22

**Authors:** Xu-feng Chen, Cong-ju Wang, Li-yuan Han, Xin Zhang, Chang Shu, Hao-yu Dong, Ya-na Ma, Bo-ya Zhang, Xu Guo, Hong-peng Sun, Gui-zhen Cao

**Affiliations:** ^1^Department of Rehabilitation Medicine, Ningbo No.2 Hospital, University of the Chinese Academy of Sciences, 315010 Ningbo, Zhejiang, China; ^2^Centers for Disease Control and Prevention of Suzhou High-tech Zone, 215000 Suzhou, Jiangsu, China; ^3^Department of Global Health, Ningbo Institute of Life and Health Industry, University of Chinese Academy of Sciences, 315010 Ningbo, Zhejiang, China; ^4^Department of Radiation Oncology, Affiliated Hospital of Guilin Medical University, 541001 Guilin, Guangxi, China; ^5^Tianjin Cerebral Vascular and Neural Degenerative Disease Key Laboratory, Tianjin Neurosurgery Institute, Tianjin Huanhu Hospital, 300060 Tianjin, China; ^6^Department of Child Health, Jiangsu Key Laboratory of Preventive and Translational Medicine for Geriatric Diseases, School of Public Health, Soochow University, 215123 Suzhou, Jiangsu, China

**Keywords:** adulthood, group-based trajectory modeling, hypertension, longitudinal cohort study, physical activity (PA)

## Abstract

**Purpose:**

We aimed to characterize physical activity (PA) trajectories across adulthood and to estimate their association with incident hypertension risk.

**Methods:**

Data were obtained from the China Health and Nutrition Survey (CHNS) conducted during 2004–2011. Group-based trajectory modeling (GBTM) was used to identify distinct groups of PA trajectories. The Cox proportional hazards model was used to investigate the association.

**Results:**

A total of 11,162 participants whose PA was repeatedly estimated by self-report from questionnaires two to four times in the CHNS were included in our study. During the 5.4 years of follow-up, 3824 incident hypertension cases were identified. Five distinct PA trajectories were identified in men: light and slight decline, light and gradual decline then sharp rise, light to medium-heavy then decline, medium-heavy and gradual decline, and heavy and sharp decline. Two distinct PA trajectories were identified in women: light and stable, and medium and gradual decline. The PA trajectory of medium-heavy and gradual decline was significantly associated with decreased risk of hypertension in men, with the hazard ratios and 95% confidence intervals (CI) being 0.80 (0.63, 0.99), 0.74 (0.59, 0.93), 0.76 (0.60, 0.96), and 0.70 (0.55, 0.88) in models 1–4, respectively.

**Conclusions:**

Our study identified five distinct long-term PA trajectories in men and two distinct trajectories in women. The PA trajectory of medium-heavy PA in early adulthood followed by gradual decline was found to be significantly associated with a decreased risk of hypertension in later life in men.

## 1. Introduction

The prevalence of hypertension in China has been increasing dramatically [1]. Its prevalence in adults was 27.9% in 2015, an increase of 9.1% compared with the prevalence reported in 2002 by a national survey [2]. Compelling evidence suggests that hypertension contributes greatly to cardiovascular and cerebrovascular diseases [3, 4]. The alarming rise in the prevalence of hypertension and its subsequent complications indicates urgent need to prevent hypertension.

As a modifiable component of lifestyle, regular moderate-intensity physical activity (PA) has been confirmed to be negatively correlated with the occurrence of hypertension [5, 6, 7, 8]. In addition, regular moderate-to-vigorous PA and leisure-time PA are also related to decreased risk of incident hypertension [9]. However, the PA–hypertension link over the course of life has not been well characterized.

To date, most studies have focused on the measurement of PA at a single time, ignoring the dynamic PA changes throughout lifetime [9, 10]. As PA varies over the course of life [11], assessing within-person trajectories of PA over time would better characterize the association between PA and diseases. The existing literature on PA trajectories is mainly focused on cardiovascular disease (CVD), pancreatic cancer, and physical functioning [12, 13, 14, 15], with limited investigations of the relationship between long-term PA trajectories and incident hypertension risk, especially in the Chinese population.

Using repeated measurements of PA taken two to four times during 2004–2011, we aimed to identify the long-term PA trajectories in a national representative sample of adults (18–63 years at baseline and 25–70 years at follow-up), and estimate their associations with incident hypertension using group-based trajectory modeling (GBTM).

## 2. Methods

### 2.1 Study Population

The China Health and Nutrition Survey (CHNS) is a national, representative study aimed at exploring the impact of social-economic transformation on Chinese health and nutrition [16]. It includes multiple samples and cohorts over nine rounds of surveys in nine provinces and three megacities between 1989 and 2011 [17]. The initial round of the CHNS was conducted in 1989, with nine follow-up rounds in 1991, 1993, 1997, 2000, 2004, 2006, 2009, 2011, and 2015. More details of the study design, sampling method as well as eligibility criteria have been published and updated recently [18].

**Fig. 1.**
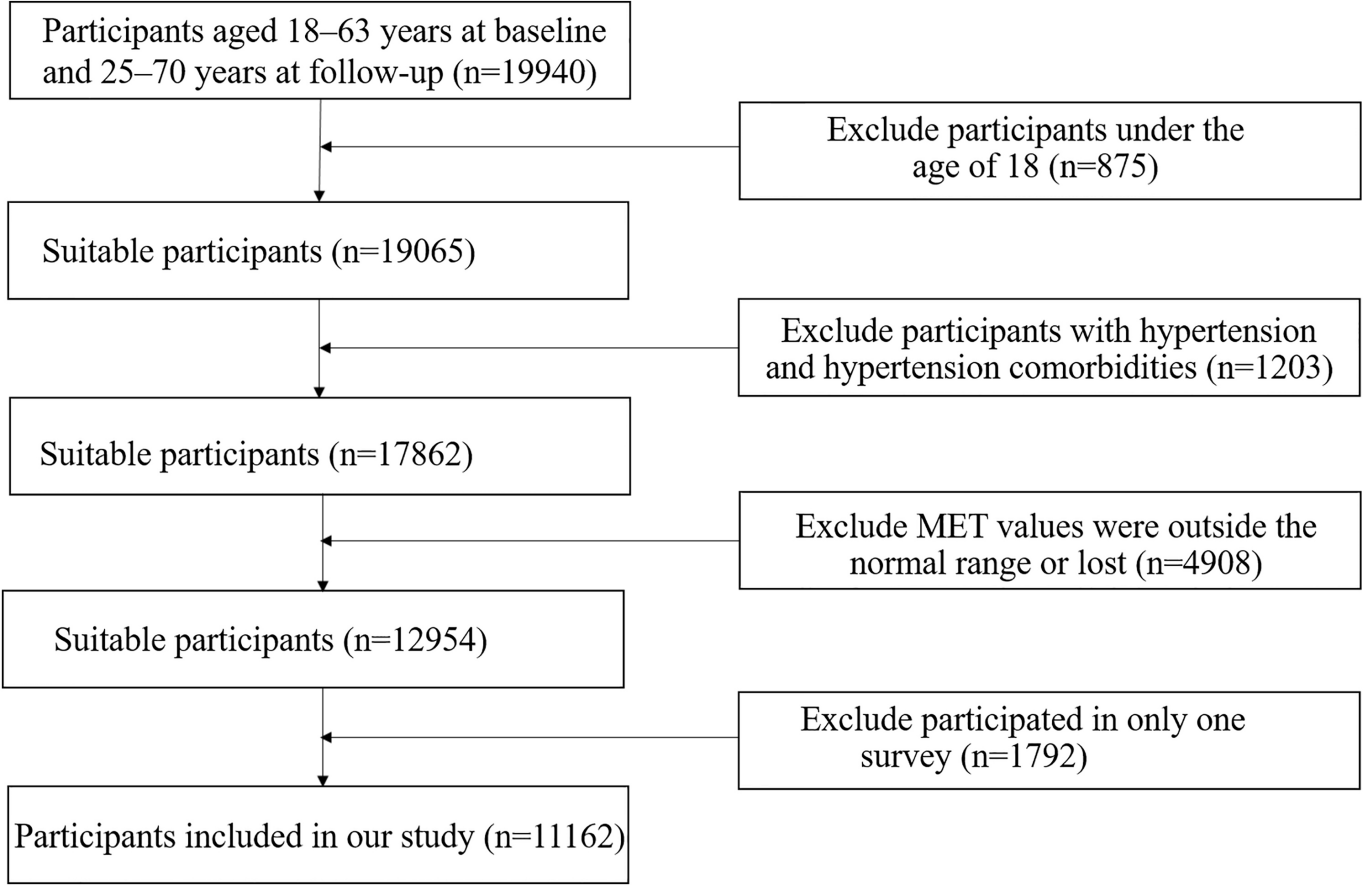
**The Flow-chart of the selection process for participants**.

As the measurements for adult sedentary leisure time were available after the 2004 survey, the present study included >18–year-old adults from the four surveys conducted between 2004 and 2011 (2004, 2006, 2009, and 2011). Our study included participants aged 18–63 years at baseline and 25–70 years at follow-up. The following participants were excluded: 875 participants under the age of 18 and 1203 participants with hypertension and hypertension comorbidities (2004 survey), 4908 participant whose metabolic equivalent of energy (MET) values were outside the normal range or lost, and 1792 individuals who participated in only one survey. A total of 11,162 participants with PA measurements available from two to four surveys were included in our study. Dtailed screening process was shown in Fig. 1. In 2004, 2006, 2009, and 2011 survey years, the number of participants was 8293, 8924, 9211, and 8424, respectively.

### 2.2 PA Measurement

In each survey, self-reported PA was collected using a standardized questionnaire [19]. Participants were surveyed on the frequency of participation and time spent in different types of PA, including occupational and domestic activities (such as cleaning, cooking, or washing), leisure activities (various forms of sports), travel activities, and sedentary leisure activities (such as sleeping, watching TV, reading, writing or drawing, playing video games or computer games, and browsing or chatting online). The intensity of each activity was expressed as MET, MET stands for metabolic equivalent of task, with one MET defined as the ratio of a person’s working metabolic rate to resting metabolic rate [20]. Vigorous activities (≥6 METs) included running, ball sports, bicycling, dance or wushu classes, and other strenuous exercise. Moderate-intensity activities (3–5 METs) included walking, driving, and doing housework. Light activities (0.9–3 METs) included sleeping, watching TV, reading, and other sedentary activities [21].

The PA level (MET-minutes/week) is the product of the specific MET values multiplied by the time spent in each activity [22]. We multiplied the number of minutes spent in each activity by the METs of the activity to calculate each PA score (MET-minutes/week), and defined the total PA score (MET-minutes/week) as the sum of METs for all activities [22]. The total PA score ranged from 3024 to 51,627 (MET-minutes/week). The complete questionnaire and scoring system used to calculate the total PA score (MET-minutes/week) has been reported in detail elsewhere [19].

### 2.3 Assessment of Incident Hypertension

Self-reported history of hypertension diagnosis and/or consumption of antihypertension medication at baseline is defined as having hypertension [23]. The incident hypertension cases in the 2006, 2009, and 2011 survey years were collected.

### 2.4 Other Measurements

Information on age; body mass index (BMI); carbohydrate, energy, fat and protein intake; urbanization index; education; smoking; drinking; and urban or rural status was collected through a questionnaire in all surveys. Doctors used standard protocols to measure height and weight to the nearest 0.1 kg and 0.1 cm, respectively. BMI was calculated by dividing the weight (kg) by the square of the height (m).

The researchers took 12 milliliters of blood from participants who had fasted for one night. The fasting plasma glucose (FPG), hemoglobin A1c (HbA1c), high-sensitivity C-reactive protein (hs-CRP), uric acid (UA), triglyceride (TG), and high-density lipopolysaccharide-cholesterol (HDL-C) levels were estimated.

Each of these variables (except classification variable) had small numbers of missing values: the largest number was 703 for BMI, while the proportion of missing values to the total number of people was only 6.3%. As the missing data accounted for only a small amount of overall data, we filled in missing numeric variables with averages.

### 2.5 Statistical Analysis

GBTM was used to define the longitudinal discrete trajectories of PA over the participants’ life course by SAS PROC TRAJ [24], which is available at www.andrew.cmu.edu/user/bjones/ [25]. Model fit was based on the bayesian information criterion (BIC), whereby the model with the lower BIC was favored [26].

Participant-years of follow-up were calculated from the date of the initial baseline interview until the date when participants were diagnosed with hypertension, the date of death, or the end of follow-up, whichever occurred first.

Distributions of covariates at baseline for each PA trajectory group membership were recorded. Categorical variables were described as percentages (%) and were compared using chi-square tests. Continuous variables were described as the mean ± standard deviation and were compared using one-way analysis of variance. A generalized linear model was used to test differences across PA trajectories.

A Cox proportional hazards model with hazard ratio (HR) and 95% confidence intervals (CI) was used to investigate the relationship between the trajectory group membership and the incident of hypertension. Model 1 was adjusted according to age. Model 2 was adjusted according to smoking and drinking status, degree of education, urban and rural, and province. Model 3 was further adjusted according to BMI. Model 4 was further adjusted according to protein, energy, fat and carbohydrate intake. Sensitivity analysis excluding participants with hypertension during the first two years of follow-up was conducted to assess whether the results were affected by reverse causation.

## 3. Results

### 3.1 Baseline Characteristics across Different PA Trajectories

A total of 11,162 participants (5368 men and 5794 women) from the 2004–2011 surveys were included in the analyses (Table 1). Table 1 presents the baseline characteristics across different PA trajectories in men and women. The average age of all participants was 38 ± 9.9 years, and those of men and women were 38 ± 9.1 and 37 ± 6.7 years, respectively. During the mean follow-up duration of 5.4 years, 3824 incident hypertensive cases were identified. Of these, 1076 (28%) cases were identified in the 2006 survey year, 1323 (35%) in the 2009 survey year, and 1425 (37%) in the 2011 survey year.

**Table 1.** **Baseline characteristics across different trajectories of physical activity in men and women**.

Baseline variables	Trajectories in men						Trajectories in women		
	Group 1 (n = 4362)	Group 2 (n = 17)	Group 3 (n = 260)	Group 4 (n = 495)	Group 5 (n = 234)	*p* value	Group 1 (n = 5123)	Group 2 (n = 671)	*p* value
Age (years)	40 (12)	45 (8.0)	40 (10)	31 (7.6)	36 (7.9)	<0.01	39 (13)	35 (9.0)	<0.01
BMI (kg/$m^{2}$)	22 (3.1)	22 (2.3)	22 (2.6)	22 (2.8)	22 (2.7)	<0.01	22 (3.2)	22 (2.7)	<0.01
Energy (kcal)	2559 (819)	2377 (584)	2894 (722)	2785 (814)	2905 (685)	<0.01	2218 (687)	2534 (676)	<0.01
Carbohydrate (g)	392 (147)	392 (122)	493 (162)	481 (169)	496 (147)	<0.01	343 (130)	436 (133)	<0.01
Fat (g)	72 (45)	56 (36)	64 (41)	57 (33)	61 (36)	<0.01	56 (34)	67 (30)	<0.01
Protein (g)	78 (38)	69 (21)	79 (26)	80 (27)	82 (28)	0.16	67 (30)	71 (25)	<0.01
Urbanization Index	74 (17)	65 (15)	57 (14)	60 (15)	55 (13)	<0.01	74 (17)	56 (13)	<0.01
High school education [n (%)]	1218 (28)	2 (12)	24 (9.3)	55 (11)	19 (8.1)	<0.01	1093 (21)	38 (5.7)	<0.01
Smoking [n (%)]	1652 (38)	4 (24)	92 (35)	164 (33)	76 (32)	0.04	185 (3.6)	29 (4.3)	0.24
Drinking [n (%)]	1841 (42)	4 (24)	102 (39)	158 (32)	76 (32)	<0.01	419 (8.2)	31 (4.6)	<0.01
Rural area [n (%)]	2641 (61)	14 (82)	223 (85)	400 (81)	201 (86)	<0.01	3179 (62)	581 (87)	<0.01
FPG (mmol/L)	5.6 (1.7)	5.0 (0.5)	5.3 (1.2)	5.3 (1.5)	5.4 (1.8)	<0.01	5.4 (1.4)	5.2 (1.1)	<0.01
${\mathrm{HbA}}_{\text{1c}}$ (%)	5.7 (1.0)	5.7 (0.5)	5.7 (0.9)	5.6 (0.9)	5.6 (0.7)	0.36	5.6 (0.9)	5.6 (1.3)	0.87
TG (mmol/L)	1.9 (1.8)	1.5 (0.8)	1.4 (1.3)	1.6 (1.5)	1.6 (1.3)	<0.01	1.6 (1.3)	1.5 (1.1)	0.01
HDL-C (mmol/L)	1.4 (0.5)	1.5 (0.4)	1.5 (0.4)	1.5 (0.6)	1.5 (0.7)	<0.01	1.5 (0.5)	1.5 (0.4)	0.42
UA (umol/L)	361 (118)	334 (100)	322 (87)	339 (86)	332 (89)	<0.01	269 (80)	256 (75)	<0.01
HS-CRP (nmol/L)	3.0 (8.3)	2.4 (2.1)	2.4 (6.5)	2.9 (14)	2.4 (6.0)	0.83	2.4 (9.8)	2.6 (7.6)	0.74

Continuous data were expressed as mean (SD); categorical data were expressed as n (%). BMI, Body mass index; FPG, Fasting plasma glucose; ${\mathrm{HbA}}_{\text{1c}}$, Hemoglobin $A_{\text{1c}}$; TG, Triglyceride; HDL-C, High density liptein cholesterol; UA, Uric acid; HS-CRP, high-sensitivity C-reactive protein. *p* represents the between-group difference between groups of different physical activity trajectories in men and women, respectively.

At baseline, age, BMI, carbohydrate, energy, fat and protein intake, urbanization index, education level, smoking, and drinking were significantly different between different PA trajectories in both men and women (*p <* 0.05). Among men, compared with the reference group (group 1), the other four trajectory groups showed lower FPG, TG, and UA levels but higher HDL-C levels at baseline (all,* p <* 0.05). Among women, compared with the reference group (group 1), the FPG, TG, and UA levels were lower in the trajectory group 2 at baseline (all, *p *< 0.05). In addition, among men, compared with group 1, group 4 showed lower average age (31 ± 7.6 years), lower fat intake, lower smoking rate, lower drinking rate, and higher protein intake at baseline.

### 3.2 PA Trajectories over 5.4 Years of Follow-Up

**Fig. 2.**
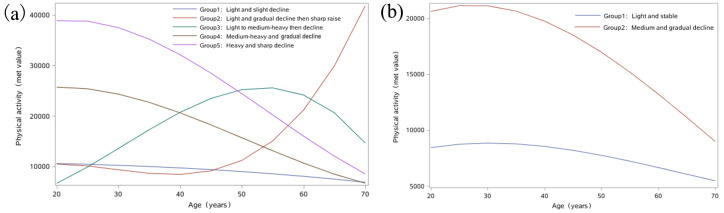
**Trajectories of physical activity in men (a) and women (b)**.

Fig. 2 shows the five distinct long-term PA trajectories in men and two distinct long-term PA trajectories in women. Detailed description of each group is given in Table 2. Among men, group 1 corresponds to men with light PA throughout adulthood (n = 4362, 81%); group 2 corresponds to men with light PA and gradual decline then sharp increase (n = 17, 0.3%); group 3 corresponds to men with light and medium-heavy PA followed by a gradual decline with age (n = 260, 4.8%); group 4 corresponds to men with medium-heavy PA in early adulthood followed by a gradual decline with age (n = 495, 9.2%); and group 5 corresponds to men with heavy PA in early adulthood followed by a gradual decline with age (n = 234, 4.4%). Among women, group 1 corresponds to women who had light PA throughout adulthood (n = 5123, 88%), and group 2 corresponds to women who had medium PA followed by a gradual decline with age (n = 671, 12%).

**Table 2.** **Different trajectories of physical activity in men and women**.

Trajectory group		Label for each group	N (%)
Men			
	Group 1	Light and slight decline	4362 (81)
	Group 2	Light and gradual decline then sharp raise	17 (0.3)
	Group 3	Light to medium-heavy then decline	260 (4.8)
	Group 4	Medium-heavy and gradual decline	495 (9.2)
	Group 5	Heavy and sharp decline	234 (4.4)
Women			
	Group 1	Light and stable	5123 (88)
	Group 2	Medium and gradual decline	671 (12)

### 3.3 Association between PA Trajectories and Incident Hypertension

The HRs and 95% CIs of the relationship between the PA trajectory stratified by gender and the incidence of hypertension are listed in Table 3. Among men, compared with the reference group (group 1), the trajectory of group 4 was significantly associated with a decreased risk of hypertension, with the HRs (95% CIs) being 0.80 (0.63, 0.99), 0.74 (0.59, 0.93), 0.76 (0.60, 0.96), and 0.70 (0.55, 0.88) in models 1, 2, 3, and 4, respectively. In the sensitivity analysis, participants who developed hypertension during the first two years of follow-up were excluded. The results in the remaining sample remained similar to those observed in the full sample, with the HRs (95% CIs) being 0.69 (0.58, 0.82), 0.61 (0.72, 0.86), 0.73 (0.57, 0.81), and 0.67 (0.48, 0.91) in models 1, 2, 3, and 4, respectively (**Supplementary Table 1**).

**Table 3.** **Associations between physical activity trajectories and risk of incident hypertension**.

Trajectories		case/N	Model 1 HR (95% CI)	Model 2 HR (95% CI)	Model 3 HR (95% CI)	Model 4 HR (95% CI)
Men						
	Group 1	1624/4362	1	1	1	1
	Group 2	9/17	1.60 (0.61, 4.24)	1.45 (0.55, 3.86)	1.52 (0.57, 4.04)	1.56 (0.58, 4.17)
	Group 3	102/260	1.11 (0.85, 1.4)	0.99 (0.76, 1.30)	1.04 (0.80, 1.37)	0.92 (0.70, 1.21)
	Group 4	115/495	0.80 (0.63, 0.99)	0.74 (0.59, 0.93)	0.76 (0.60, 0.96)	0.70 (0.55, 0.88)
	Group 5	85/234	1.22 (0.92, 1.61)	1.10 (0.83, 1.47)	1.11 (0.83, 1.49)	0.99 (0.74, 1.32)
Women						
	Group 1	1692/5123	1	1	1	1
	Group 2	197/671	1.12 (0.93, 1.35)	0.99 (0.82, 1.19)	1.03 (0.85, 1.25)	0.86 (0.71, 1.05)

Model 1 was adjusted by age; Model 2 was further adjusted by smoking, drinking, education, urban or rural status, province status based on model 1; Model 3 was further adjusted by BMI based on model 2; Model 4 was further adjusted by energy, carbohydrate, fat, and protein intake based on model 3. case/N, Number of hypertension cases/number of participants in this trajectory group. In men, group 1, light and slight decline; group 2, light and gradual decline followed by sharp rise; group 3, light to medium-heavy; group 4, medium-heavy and gradual decline; group 5, heavy and sharp decline. In women, group 1, light and stable; group 2, medium and gradual decline.

## 4. Discussion

In this national prospective study with repeated measurements of PA over the lifetime of participants, we identified five distinct long-term PA trajectory groups in men and two such groups in women. In men, we found that the trajectory group labeled as medium-heavy PA in early adulthood followed by gradual decline was significantly associated with incident hypertension risk in later life.

To date, the research on PA trajectory has focused primarily on CVD and physical functioning; for example, one study showed that a 20-year PA trajectory (moderate increase in PA level from middle age to old age) was associated with a decreased risk of mortality and CVD in later life, with an observed dose-response relationship, and that maintaining even a slight PA was helpful [12]. Another study demonstrated that compared with women in the low PA groups, those in the middle and highest PA groups had more than 5% better physical functioning performance in later life [15]. However, these findings and ours cannot be compared directly owing to the differences in study populations, study design, sample size, methodology, and follow-up time. In addition, the PA calculation in those studies only included sport/exercise and excluded domestic, travel, leisure, and sedentary activities. In contrast, our study incorporated a comprehensive calculation of PA score (MET-minutes/week), and complemented the current evidence of association between PA trajectory and incident hypertension.

Among men, the majority of participants belonged to group 1 (light PA and slight decline), indicating the high prevalence of a light, persistently stable PA trajectory in adulthood. The national representative sample of our study suggests that great effort should be dedicated to promoting PA activities. The Healthy China 2030 platform advocates several strategies to promote the adoption of PA, such as formulating and implementing extensive national fitness campaigns; strengthening the integration of physical and medical pathways (publishing sports and fitness activity guidelines); and formulating and implementing physical health intervention plans for special groups (adolescents, women, the elderly, and disabled people).

Notably, group 4, labeled as medium-heavy PA followed by gradual decline, accounted for 9.2% of the study sample, and was significantly related to decreased risk of incident hypertension. Previous studies have shown that medium-heavy PA in early adulthood is associated with declined hypertension risk [27]. In addition, compared with the reference group, the participants in group 4 were younger; had lower levels of FPG, TG, and UA; had higher levels of HDL-C; had lower fat intake and more protein intake; and included fewer smokers and drinkers. All of these factors were positively correlated to the occurrence of hypertension, with their lower levels contributing to reduced hypertension risk [28]. In this study, we emphasize the importance of maintaining medium-heavy PA in early adulthood, especially for men.

Furthermore, no significant association was observed between the identified trajectory groups and incident hypertension in women. Consistent with our findings, prospective studies also reported non-significant correlation between PA and hypertension risk in women [29, 30]. Both the intensity of PA and the total amount of energy spent are lower in women than in men. Notably, compared with men, woman spend a greater proportion of time engaged in sedentary and light activities and less time engaged in more strenuous (moderate and intense) PA [31, 32]. Furthermore, women’s blood pressure is also influenced by estrogen levels, menstrual cycle, and fertility [33]. Taken together, these findings suggest that women should be the priority target for PA promotion. Our study has important public health implications. Women should be the prioritized target population for physical health interventions. Public health workers should be involved in distributing informative materials related to PA (illustrations, small foldouts, desk calendars, CD-ROMs, etc.) and organizing public awareness activities on PA lectures and health consultations. PA interventions can also be delivered through avenues such as social/familial events to enhance their effectiveness [34]. For example, people could be encouraged to establish an exercise group within the family or sign exercise contracts with each other to complete a certain amount of PA.

## 5. Strengths

To the best of our knowledge, this is the first study to identify the long-term PA trajectories in a representative national sample of Chinese adults and to investigate the effect of PA trajectory on incident hypertension risk. The strengths of our study also include the large sample size, the availability of repeated measures of PA over time, and the use of GBTM. GBTM is a powerful statistical tool that applies limited hybrid modeling and maximum likelihood estimation to determine different PA trajectories [35]. Furthermore, not only occupational but also domestic, travel, leisure, and sedentary activities were included in the calculation of total PA score (MET-minutes/week) in our study.

## 6. Limitations

It should be noted that this study has some limitations. PA was self-reported rather than objectively measured; thus, a certain degree of recall bias cannot be ruled out. In addition, the CHNS only includes the Chinese Han population, so the findings may not be generalizable to other populations. Next, our study is limited by a short follow-up time (average of 5.4 years). At the end of the follow-up period, some young participants may not have developed hypertension. However, the similar results obtained in sensitivity analysis by excluding participants who developed hypertension during the first two years of follow-up are evidence for the reliability of our findings. In addition, the BP was not measured at each interview is also a shortcoming of this study. Finally, due to the absence of the covariate (BMI, energy intake) during follow-up, the covariates at baseline were used in Model 2, 3 and 4.

## 7. Conclusions

In conclusion, we identified five distinct long-term PA trajectories in men and two distinct PA trajectories in women. The PA trajectory of medium-heavy PA in early adulthood followed by gradual decline was significantly associated with a decreased risk of hypertension in later life in men. Our study emphasizes the preventive effects of medium-heavy PA in early adulthood against incident hypertension in later life, highlighting that medium-heavy PA should be advocated in early adulthood and should be maintained throughout adulthood.

## Supplementary Material

Supplementary material associated with this article can be found, in the online version, at https://doi.org/10.31083/j.rcm2311385.
