# Supplementary material for: Associations between Physical Activity Trajectories and Incident Hypertension
Source: Rev Cardiovasc Med. 2022 Nov 22;23(11):385. doi: 10.31083/j.rcm2311385 (PMC11269057; doi:10.31083/j.rcm2311385)
Supplement: Supplementary file 1 [file 2153-8174-23-11-385-s1.docx]

**Table S1.** Associations between physical activity trajectories and risk of incident hypertension after

| Trajectories | | case/N | Model 1  HR (95% CI) | Model 2  HR (95% CI) | Model 3  HR (95% CI) | Model 4  HR (95% CI) |
| --- | --- | --- | --- | --- | --- | --- |
| Men  Group 1 | | 1106/4362 | 1 | 1 | 1 | 1 |
| Group 2 | 5/17 | | 1.55  (0.49,3.21) | 1.29  (0.57,1.89) | 1.90  (0.71,4.02) | 1.88  (0.99,3.14) |
| Group 3 | 89/260 | | 1.66  (0.54,1.83) | 0.67  (0.71,2.02) | 1.32  (0.77,1.81) | 1.02  (0.46,1.57) |
| Group 4 | 98/495 | | 0.69  (0.58,0.82) | 0.61  (0.72,0.86) | 0.73  (0.57,0.81) | 0.67  (0.48,0.91) |
| Group 5 | 81/234 556 | | 1.19  (0.90,1.35) | 1.55  (0.82,1.88) | 1.09  (0.63,1.40) | 0.87  (0.78,1.33) |
| Women  Group 1 | | 1229/5123 | 1 | 1 | 1 | 1 |
| Group 2 | | 140/671 | 1.46  (0.73,1.67) | 0.92  (0.83,1.44) | 1.07  (0.99,1.53) | 0.64  (0.69,1.36) |

Model 1 was adjusted by age.

Model 2 was further adjusted by smoking, drinking, education, urban or rural status, province status based on model 1.

Model 3 was further adjusted by BMI based on model 2.

Model 4 was further adjusted by energy intake, carbohydrate intake, fat intake, protein intake based on model 3.

^a^ case/N: Number of hypertension cases/number of participants in this trajectory group

In men, group 1: light and slight decline; group 2: light and gradual decline then sharp raise; group 3: light to medium-heavy; group 4: medium-heavy and persistent decline; group 5: heavy and sharp decline

In women, group 1: light and stable; group 2: medium and gradual decline

*In the sensitivity analysis, participants who developed hypertension during the first 2 years of follow-up were excluded.
